# Publisher Correction: Optimal Mass Transport with Lagrangian Workflow Reveals Advective and Diffusion Driven Solute Transport in the Glymphatic System

**DOI:** 10.1038/s41598-020-60586-2

**Published:** 2020-02-27

**Authors:** Sunil Koundal, Rena Elkin, Saad Nadeem, Yuechuan Xue, Stefan Constantinou, Simon Sanggaard, Xiaodan Liu, Brittany Monte, Feng Xu, William Van Nostrand, Maiken Nedergaard, Hedok Lee, Joanna Wardlaw, Helene Benveniste, Allen Tannenbaum

**Affiliations:** 1 Department of Anesthesiology, Yale School of Medicine, New Haven, CT USA; 2 Departments of Computer Science/Applied Mathematics and Statistics, Stony Brook University, Stony Brook, NY USA; 3 Department of Medical Physics, Memorial Sloan Kettering Cancer Center, NY, NY USA; 4 George & Anne Ryan Institute for Neuroscience, Department of Biomedical and Pharmaceutical Sciences, University of Rhode Island, Rhode Island, RI USA; 5 Center for Translational Neuromedicine, University of Rochester Medical Center, Rochester, NY USA; 6 Brain Research Imaging Centre, Centre for Clinical Brain Sciences, Dementia Research Institute at the University of Edinburgh, Edinburgh, United Kingdom

Correction to: *Scientific Reports* 10.1038/s41598-020-59045-9, published online 06 February 2020

The original version of this Article contained a typographical error in the spelling of the author Sunil Koundal, which was incorrectly given as Sunil Kounda. This has now been corrected in the PDF and HTML versions of the Article.

