# Supplementary material for: Optimal Mass Transport with Lagrangian Workflow Reveals Advective and Diffusion Driven Solute Transport in the Glymphatic System
Source: Sci Rep. 2020 Feb 6;10:1990. doi: 10.1038/s41598-020-59045-9 (PMC7004986; doi:10.1038/s41598-020-59045-9)
Supplement: Supplementary file 1 — Supplementary information. [file 41598_2020_59045_MOESM1_ESM.pdf]

Supplementary Information for "Optimal Mass Transport with Lagrangian Workflow Reveals Advective and Diffusion Driven Solute Transport in the Glymphatic System" by

Sunil Koundal[1o], Rena Elkin[2o], Saad Nadeem[3], Yuechuan Xue[1], Stefan Constantinou[1], Simon Sanggaard[1], Xiaodan Liu[1], Brittany Monte[1], Feng Xu[4], William Van Nostrand[4], Maiken Nedergaard[5], Hedok Lee[1], Joanna Wardlaw[6], Helene Benveniste[1*+] and Allen Tannenbaum[2*+]

[*]Corresponding Author
[o]These authors contributed equally to this work
[+]Co-Senior Authors

(1) Department of Anesthesiology, Yale School of Medicine, New Haven, CT, USA
(2) Departments of Computer Science/Applied Mathematics and Statistics, Stony Brook University, Stony Brook, NY, USA
(3) Department of Medical Physics, Memorial Sloan Kettering Cancer Center, NY, NY, USA
(4) George & Anne Ryan Institute for Neuroscience, Department of Biomedical and Pharmaceutical Sciences, University of Rhode Island, RI, USA
(5) Center for Translational Neuromedicine, University of Rochester Medical Center, Rochester, NY, USA
(6) Brain Research Imaging Centre, Centre for Clinical Brain Sciences, Dementia Research Institute at the University of Edinburgh, Edinburgh, United Kingdom

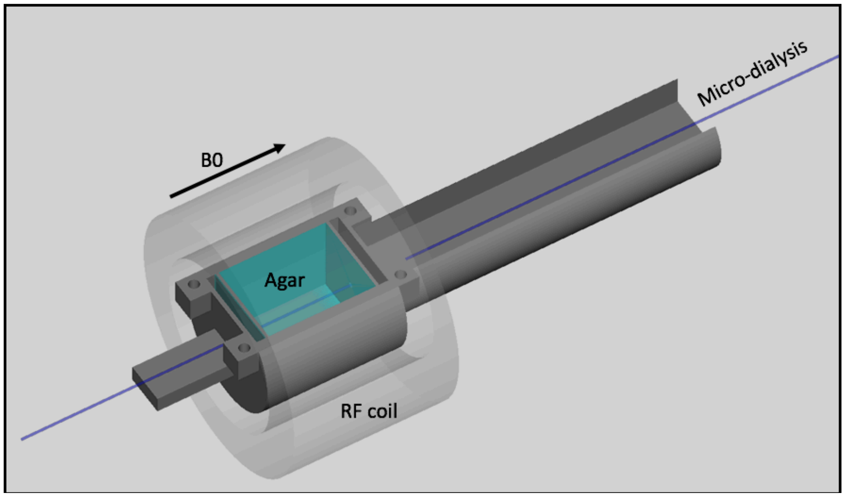

Supplementary Fig. 1: Diffusion phantom with microdialysis tube for validating diffusion constraint in the rOMT formulation. We validated that the rOMT algorithm captures Fick's law using a custom built 'diffusion' phantom with a microdialysis membrane. A linear designed microdialysis probe (outer diameter 260µm, molecular weight cut off 55kDa, CMR 31) was inserted through the center of a 3D printed, MR compatible holder containing 0.5% agar (20x20x30 mm). The phantom was placed in the 9.4T MRI scanner equipped with 40 mm ID RF transmit/receive coil and the microdialysis membrane was perfused for 43 min (108 µl) with Gd-DOTA (12 mM) at 2.5 µl/min using a micro-infusion pump after which Gd-DOTA solutes moved into the agar medium driven by diffusion. Pre- and post-contrast DCE-MRI were acquired using the same parameters as for the *in vivo* study. A total of 140 frames were collected at an interval of 2.5 min per frame under constant temperature (21- 22°C).

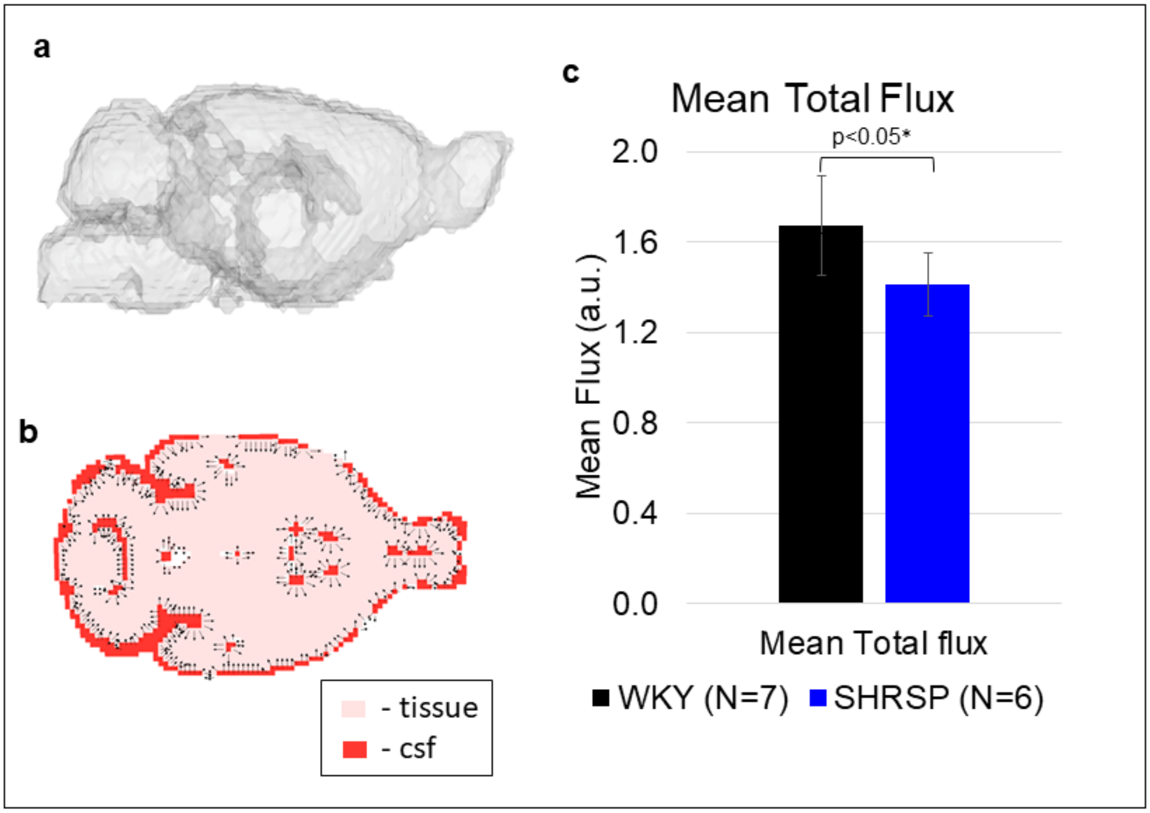

Supplemental Fig. 2 rOMT relation to compartmental models for directional tracer movement from CSF into the tissue. (a) 3D rendering of boundary B of tissue compartment C. (**b)** Representative slice of the whole brain showing the CSF compartment in red and the tissue compartment (gray matter and white matter) in pink. Normal vectors (of unit length) pointing from the CSF compartment into the tissue compartment are shown along the CSF-tissue boundary as black arrows. (**c)** Mean (advective + diffusive) flux that moved from CSF into the tissue compartment is significantly higher in normotensive (WKY) than hypertensive (SHRSP) cases.

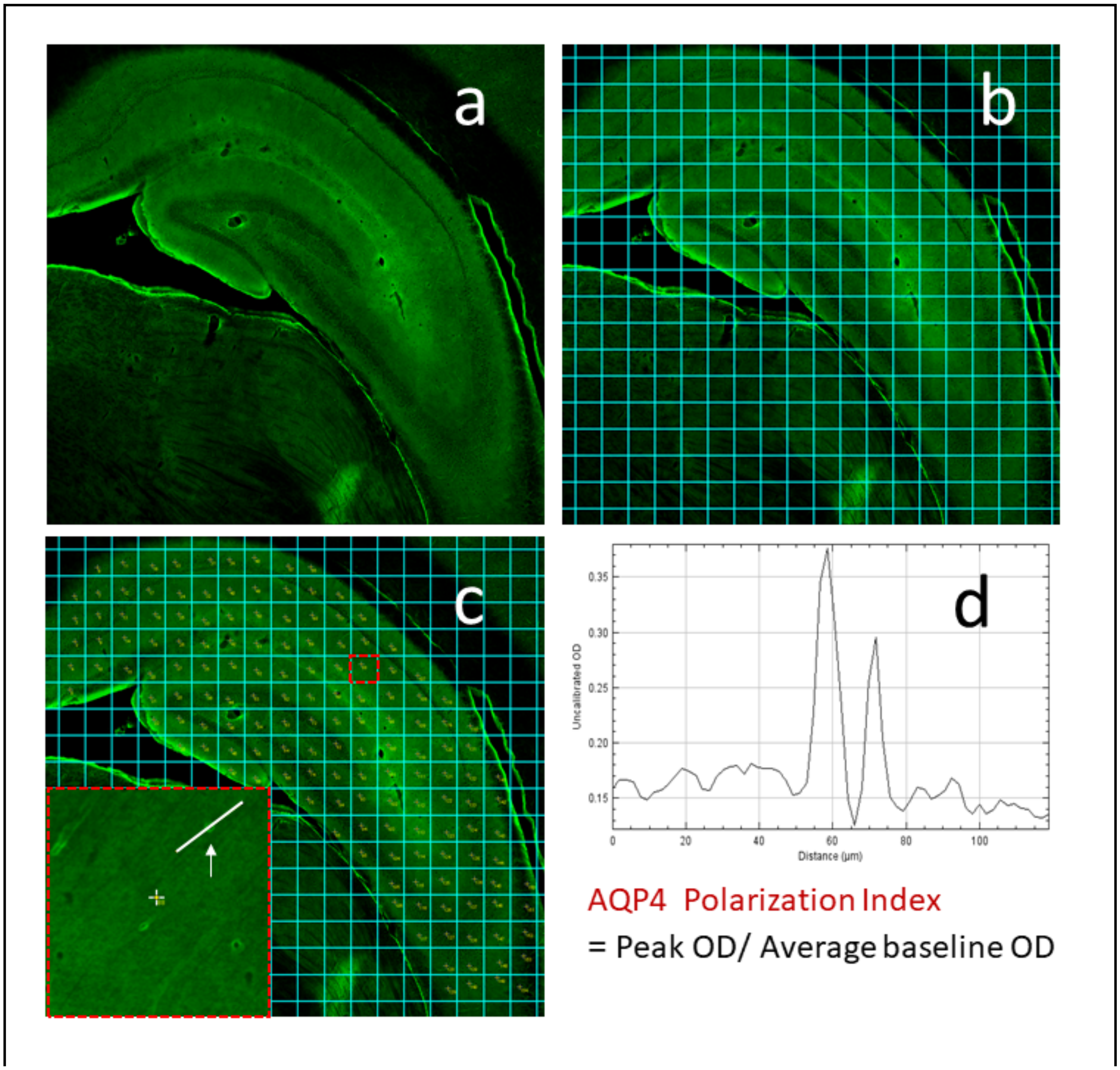

Supplementary Fig. 3: Measuring AQP4 polarization index in WKY and SHRSP rats. (a) AQP4 immunofluorescence at the level of ventral hippocampus from a WKY rat. **(b)** For quantification of AQP4 polarization, a uniform, 154 box grid was overlaid using ImageJ (Image J 1.52i). **(c)** 10 and 15 random numbers were selected from the 154-box grid for measurement of small vessels and capillaries, respectively. The arrow points to the linear ROIs crossing the lumen of the small vessel of interest in the grid of interest. **(d)** The optical density profile from the ROI in (c) is displayed along with the calculated polarization index. Specifically, for polarity calculation, the peak intensity of the vascular end-feet was divided by the average of the baseline.

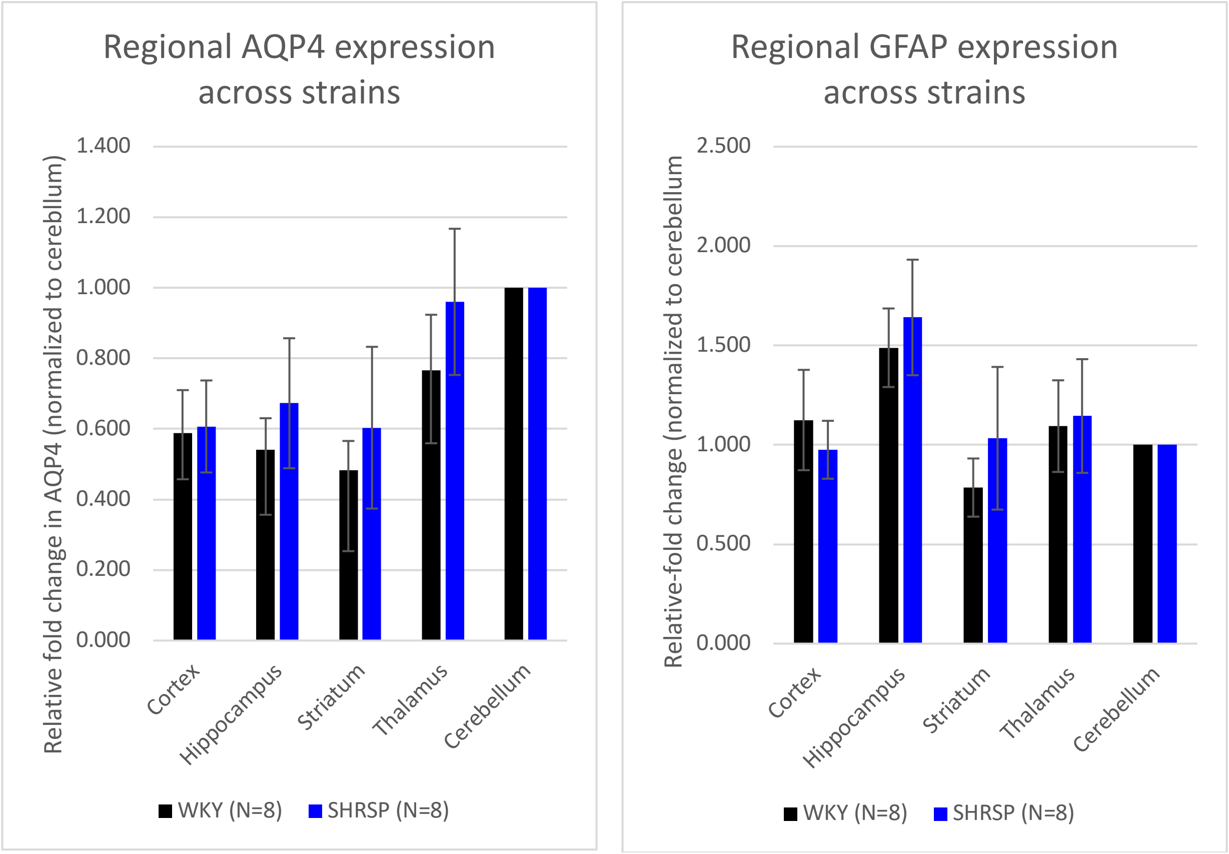

Supplementary Fig. 4: qPCR analysis of GFAP and AQP4 expression in WKY and SHRSP rats. qPCR analysis of AQP4 (left) and GFAP (right) mRNA expression in WKY (n=8) and SHRSP (n=8) rats assessed in cortex, hippocampus, striatum and thalamus. Relative fold change values were calculated by normalizing to the cerebellum. Data are expressed as mean relative fold change values ± standard deviation. No significant differences were found between regional AQP4 and GFAP mRNA expression across strains. All statistical analyses were performed using Addinsoft (2019). XLSTAT statistical and data analysis solution. Boston, USA. https://www.xlstat.com.

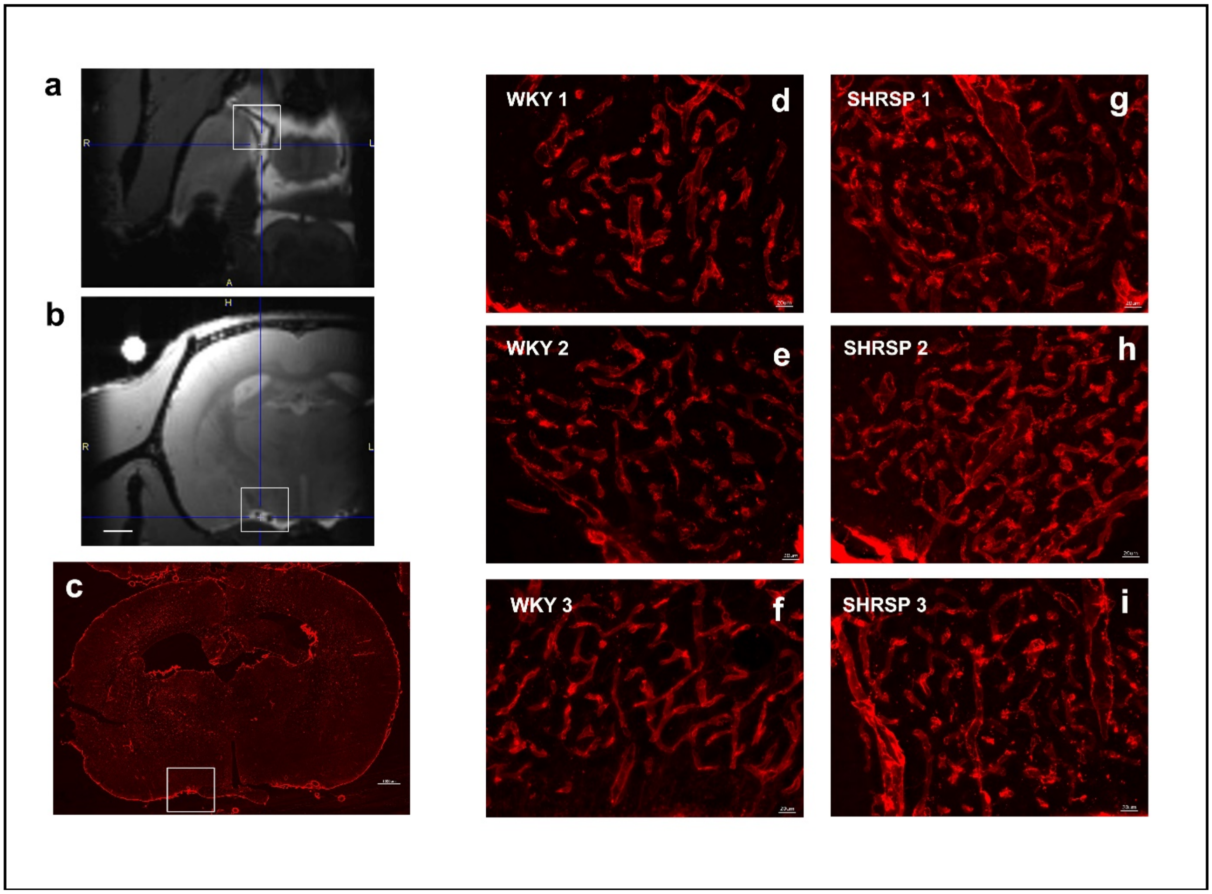

Supplementary Fig. 5: Exploration of the vasculature and microvasculature in WKY and SHRSP at the level of circle of Willis. (a, b) Contrast enhanced MRIs from a WKY rat at the level of the circle of Willis near the take off of the middle cerebral artery (MCA). Contrast in the perivascular space along the circle of Willis and middle cerebral artery is evidenced as a high intensity signal along the vasculature (black signal). **(c)** Collagen IV immunofluorescence of a WKY rat. Insert shows the area of interest near the large vessels at the ventral surface of the brain. **(d-f)** Representative confocal montages of collagen IV labeling from three different WKY rats at the site highlighted by the white box in (c). Smaller networks of capillaries (7-10 µm in diameter) were observed at the base of the brain surrounding penetrating arteries and appear interweaved into a small network. **(g-i)** In corresponding images from the SHRSP rats, collagen IV labeling showed the capillary networks (a.k.a. rete) were distributed over slightly larger areas around penetrating arteries. Further, the capillary networks appeared denser and the individual vessels were larger (10-20 µm in diameter). We speculate that the microvascular network remodeling might have affected the PVS transport from the circle of Willis in SHRSP compared to WKY rats.

Supplemental Movie 1. Regularized OMT model (rOMT) captures diffusive agar phantom behavior. rOMT-returned interpolated images (right) are consistent with DCE-MRI phantom data (left). rOMT successfully models diffusive transport from regions of higher intensity to regions of lower intensity in accordance with Fick's law, shown by diffusive flux vectors in green. See Movie1.mov.

Supplementary Movie 2: Contrast-enhanced dynamic MRI from normal WKY rat showing signal changes along the middle cerebral artery over ≈3hr CSF Gd-DOTA circulation. See Movie2.mpg.

Supplementary Table 1: Physiological data and anesthetics

Experiment	Parameter	Animals anesthetized?	WKY (N=11)	SHRSP (N=11)	p-value
**Non-invasive Blood pressure**	Systolic blood pressure (mmHg)	No	116 ± 14	150 ± 23	**0.0004**
	Diastolic blood pressure (mmHg)	No	85 ± 11	98 ± 21	0.151
	Pulse pressure (mmHg)	No	32 ± 9	51 ± 13	**0.001**
**Experiment**	**Parameter**	**Animals anesthetized?**	**WKY (N=6)**	**SHRSP (N=5)**	**p-value**
**Invasive blood pressure**	Mean arterial blood pressure (mmHg)	Yes	76 ± 14	123 ± 25	**0.004**
**Experiment**	**Parameter**	**Animals anesthetized?**	**WKY (N=11)**	**SHRSP (N=10)**	**p-value**
**Glymphatics Experiments**	Mean heart rate (bpm)	Yes	221 ± 20	235 ± 14	0.049
	Mean respiratory rate (breath/min)	Yes	45 ± 6	46 ± 7	0.650
	Mean infusion rate of dexmedetomidine* (ml/hr)	N/A	0.10 ± 0.03	0.10 ± 0.03	0.748
	Mean inspiratory isoflurane concentration (%)	N/A	1.1 ± 0.2	1.0 ± 0.2	0.932

Data are mean ± SD. *Concentration of dexmedetomidine used for infusion: 0.025mg/ml.
